# Efficacy and Risk Factor Analysis of DBT Therapy for PTSD-Related Symptoms in Mainland Chinese College Students Based on Data Mining

**DOI:** 10.1155/2022/3829623

**Published:** 2022-09-22

**Authors:** Chenyang Xiao

**Affiliations:** The University of Hong Kong, Hong Kong, China

## Abstract

Posttraumatic stress disorder (PTSD) is a mental disorder characterized by a delayed onset and long-lasting psychiatric disorder in an individual due to unusual threatening or catastrophic stressful events, characterized by repeated experiences of the situation, avoidant behaviors, emotional numbness, hypervigilance, and other mental symptoms. It seriously affects the occupational, psychological, and social functions of the human body, leads to a decrease in the quality of life, and brings a greater economic burden to the patients themselves, their families, and the society. It has attracted widespread attention worldwide. Due to social transformation and fierce competition, college students are increasingly exposed to various stressful or traumatic events, and PTSD is becoming more and more obvious. Therefore, this study took a university student as the research object, analyzed the risk factors of PTSD, and used the method of data mining to analyze the effectiveness of DBT therapy and completed the following work: (1) this paper introduces the research status of PTSD pathogenesis at home and abroad and expounds the treatment methods and research results of DBT. (2) The basic principle of BPNN is introduced, the weight and threshold of BPNN are screened by genetic algorithm, and the best weight and threshold after screening are given to BPNN. A GA-BP model is constructed to improve the learning quality of BPNN. (3) The optimal parameters of the model are selected through experiments, and the model is verified by the collected data. The results show that the model has superiority in evaluating the effectiveness of DBT therapy. Then, it was proved by experiments that DBT therapy has a good effect in the treatment of PTSD. Finally, the influencing factors of PTSD were analyzed one by one through the experimental results.

## 1. Introduction

The term PTSD is used to describe a mental illness in which people have a delayed onset and long-term maintenance of symptoms after experiencing a traumatic incident. Its clinical manifestations include three core symptom groups: intrusive symptoms, avoidance symptoms, and increased irritability [1, 2]. It is the most common kind of stress mental illness, characterized by atypical responses to stressful events such as significant catastrophes (such as wars and terrorist attacks), traffic accidents, and severe illnesses (such as cancer) [3]. With the increase of unexpected events such as war, violence, major accidents, and natural disasters, especially the sudden new crown epidemic, the incidence of PTSD has increased year by year [4]. Due to its high prevalence, extended duration, and challenging treatment, PTSD is an essential stress-related psychiatric condition. PTSD adversely affects the physical and mental health of sufferers. At present, some achievements have been made in the mechanism research and clinical research, and it needs to be further strengthened. The disease is currently one of the key difficulties in psychiatry, mainly determined by its own characteristics of posttraumatic stress disorder. There are two characteristics: uniqueness and complexity. In view of the uniqueness and complexity of PTSD, some progress has been made in the study of the pathological mechanism of PTSD by animal studies and human studies, and three main theoretical systems have been established. There are three main theories of posttraumatic stress disorder: conditioned reflex and conditioned reflex generalization theory, cognitive theory, and social-individual theory [5–7]. All three theories have been preliminarily proved by animal studies or human studies that there are plastic changes in brain structure and brain function. According to the principles of evidence-based medicine, these three theories have provided a certain theoretical basis for clinical work and crisis intervention work. Therefore, various treatment methods such as cognitive behavioral therapy, exposure therapy, transcranial magnetic stimulation therapy, realistic imagination therapy, eye movement therapy, and drug therapy for trauma were carried out [8–10]. Some treatment methods choose a single treatment, and some use a combination of two treatments. There are long-term treatments and short-term treatments. In clinical cases, the trauma experienced is from violent events, after wars, after natural disasters, etc. These therapies have been used in several clinical research and practises and have been successful in treating posttraumatic stress disorder in adults and adolescents [11–13]. There is also more research into clinical studies and interventions, with studies proving that school-based intervention models for students also have great benefits. Participation of family members in treatment and intervention is more effective for those with PTSD than receiving treatment and intervention independently [14, 15]. The results of many studies also strongly demonstrate the benefits of emergency intervention after trauma [16]. Studies have shown that cognitive therapy is more effective than early exposure intervention [17]. Dialectical Behavior Therapy (DBT) is one of the most well-known psychotherapies for balancing “acceptance and change.” DBT was originally used to treat patients with borderline personality disorder who had low self-efficacy and difficulty managing their emotions. Now people are increasingly using DBT technology to treat PTSD patients and have achieved remarkable results [18]. This is especially true for patients with multiple childhood traumatic experiences and extensive problems with emotional management. DBT provides a clear, phased approach to therapy, with the focus and content of each phase corresponding to the stage of the patient's psychological disorder. DBT generally divides the treatment process into three stages [19]. The first stage is to help patients eliminate or reduce problem behaviors that are dangerous to them by teaching them some techniques to control their behavior and manage their emotions. The second phase occurs when patients have learned and are able to apply those techniques widely. The second stage involves teaching patients some dialectical-oriented behavioral change techniques and some acceptance strategies to confront patients with past emotional processes, as well as symptoms of denial, embarrassment, self-blame, intrusive memories, and flashbacks. Help patients accept and integrate their past and cope with posttraumatic stress. The third stage is when the patient applies the previously acquired skills to achieve their goals and increase their self-efficacy. Often DBT therapists will also conduct a diagnostic and behavioral assessment of the patient before formal initiation of treatment and establish a treatment contract with the patient after the patient is fully acclimated to the treatment approach. Today's society is changing with each passing day. College students are facing social transformation, increasingly fierce competition, and employment pressure and are exposed to various stressful or traumatic events. Moreover, as a special group, college students have not yet fully matured psychologically, and the incidence of PTSD has become more and more obvious [20, 21]. Therefore, this study takes a university student as the research object, preliminarily analyzes the epidemiological characteristics of college students' PTSD, and further explores its influencing factors and explores the effectiveness of DBT therapy, as a theoretical framework for preventing and treating college students' posttraumatic stress disorder and other psychiatric problems.

## 2. Related Work

Behavioral therapy has held an important place in the field of psychotherapy since its introduction in the 1920s. The American psychology community generally believes that the emergence and occurrence of behavioral therapy has gone through four stages [22]. The first stage is the formation period of the classical theory of behaviorism, which established the basic theory of behaviorism and laid the foundation for the development of behavioral therapy later. The second stage was in the 1960s and 1970s, when behaviorist theory was systematically applied to solve practical human psychological problems. The first behavioral therapies appeared, such as token reward and punishment, positive reinforcement, punishment, extinction, and system desensitization. This generation of therapy typically employs simple and straightforward corrections in the process, focusing on immediate relief of the patient's symptoms. The third stage is the combination of behavioral therapy and cognitive psychology. A group of cognitive psychologists was born and began to solve psychological problems with new behavioral therapy, namely, cognitive therapy. The most representative of them are rational emotion therapy and Beck cognitive therapy which is called “second-generation behavioral therapy.” The fourth stage was in the late 1980s, when researchers incorporated traditional cognitive behavioral therapy into emerging methods and discoveries. This stage of therapy no longer focused on the form of treatment, but on the situational and functional sensitivities of psychological phenomena, so it is also known as situational behaviorist therapy. The therapy tends to emphasize situational and experiential behavioral change strategies, looking for a broad, flexible, and effective all-round skills framework [23]. DBT is an evidence-based integrative psychosocial therapy originally developed for the treatment of borderline personality disorder. Cognitive therapy with an emphasis on emotion regulation mixed with mindfulness treatment and dialectical philosophy is offered to help individuals with borderline personality disorder better regulate and manage their emotions when faced with stressful situations and interpersonal discomfort [24]. Psychology and clinical psychology have been interested in this therapy since it was first proposed. It is now widely used to help people with a variety of mental health issues, such as anxiety disorders, depression, and eating disorders. It can also be used to help people who have difficulty controlling their emotions, whether they are adults or teenagers. An important theory on which the therapy was originally founded is the biosocial theory of emotional dysregulation in borderline personality disorder [25]. One cause of emotional dysregulation is a malfunctioning emotion regulation system in the brain, which causes people to be more sensitive to their emotions, to have more intense emotional reactions, and to be less resilient to their emotions. On the other hand, difficulty in emotion regulation can be affected by many aspects such as individual early childhood experiences and social environment [26]. Therefore, DBT is a psychotherapy with the core of solving the problem of emotional imbalance and improving the ability of emotional regulation. DBT also incorporates behaviorist theory, which holds that behavior is caused by human factors. Behavior change may be achieved through identifying the behavioral elements that lead to, maintain, and perpetuate the behaviors, breaking the connections, changing the consequences, and developing new models of the new behaviors [27]. According to this idea, DBT treatment approaches concentrate on teaching clients new skills, stress control, and self-exposure in addition to retraining their brains to think in new ways. DBT also incorporates theories of dialectical philosophy. In general terms, dialectical theory emphasizes that reality is interconnected, and that everything is composed of two opposite sides. The opposing sides are constantly changing and can change each other [28]. In DBT, the counselor usually adopts a dialectical attitude while holding opposing viewpoints and helps the client accept reality in the dialectical opposition. Therefore, dialectics is a strategy in DBT, and the main dialectics in DBT is the relationship between acceptance and change, the counselor teaches clients skills to change behavior and also tells them how to accept themselves and reality [29]. The most fundamental principle of DBT is the contradictory balance of accepting strategies and changing strategies. This principle determines its uniqueness and how it differs from traditional cognitive behavioral therapy. Acceptance strategies include total acceptance of the client's existing living conditions and behaviors, and change strategies include problem solving, changing pessimistic emotions and behaviors, and so on. This principle persists throughout the course of the treatment [30]. In terms of treatment mode, DBT includes four forms of skill training group, individual psychotherapy, telephone consultation, and therapist supervision group [31]. Skills training groups belong to group counseling, aiming at clinical treatment of mental diseases such as borderline personality disorder, depression, and anxiety. Individual psychotherapy accompanies the entire treatment process and plays a role in diagnosis, negotiation of treatment goals and steps, and promotion of treatment motivation. Standardized telephone counseling helps patients apply DBT skills in a timely manner to address impulse control problems in their lives and reduce the risk of self-harm and suicide. The therapist supervision team provides professional skills support, generally including 4–8 therapists, supervising 1–2 hours per week, to avoid the therapist's exhaustion due to long-term stress, and to improve their service motivation and competence.

## 3. Method

### 3.1. BP Neural Network

Error backpropagation is used to train BPNN, a feedforward network learning technique. Input, hidden, and output layers make up its structure. Multiple layers may be hidden under the surface. The neurons inside each layer are not linked; however, the neurons that connect the layers are. The neurons in each layer gradually advance to the output layer through the weight threshold assigned by the algorithm. When the output layer is reached and the predicted output is obtained, it is compared with the actual output value, the error is transferred in reverse, and the weight thresholds of each layer are reversely adjusted according to the size of the error, and finally the predicted output is continuously approached to the real output value. This process is the process of continuous optimization and learning. The specific algorithm is as follows.

(1) Data preprocessing normalizes the input variable *x* to [−1, 1]:
  (1) $$\begin{array}{c} n_{i}=\frac{x_{i}-x_{\min}}{x_{\max}-x_{\min}}\text{,} \end{array}$$
  where *n*_*i*_ is the normalized result of *i*, *x*_*i*_ is the input of *i*, and *x*_max_ and *x*_min_ are the maximum and minimum values of the original input variables, respectively.
(2) Forward propagation: data goes from the input layer to the hidden layer and then to the output layer. Each hidden neuron and output neuron processes its input, multiplies each input by its weight, sums its products, and passes the sum through the activation function to get the predicted output:
  (2) $$\begin{array}{c} y_{f}=f\left({net}_{i}\right)f\left(\underset{j}{\sum }w_{ij}n_{i}+b_{i}\right)\text{,} \end{array}$$
  where *f*(net_*i*_) is the activation function and the transfer threshold is [0, 1], *n*_*i*_ is the normalized result, *w*_*ij*_ is the weight of the connection from unit *i* to unit *j*, and *b*_*i*_ is the bias term.
(3) Error backpropagation: during the error backpropagation process, the neural network enters when the actual result does not match the intended outcome. The error gradient descends from the output layer to the hidden layer and the input layer, and the linked weights are updated using the gradient equation for the connected weights. Keep repeating the above steps until the error of the network falls below the target:
  (3) $$\begin{array}{c} f_{b}=-\varepsilon_{i}+\underset{j>1}{\sum }w_{ij}\delta_{i}\text{,} \end{array}$$
  where *ε* is the summation index and *j*, *i*, and *ε* are the product of errors.
(4) Training process: the term “repeated forward propagation and backpropagation” refers to the neural network model's training and learning process. Training continues until the mistake has been reduced to an acceptable level or has reached the prescribed number of repetitions, at which point the training is complete. Transfer, training, learning, and performance functions are all forms of BP neural network functions that are often used. Each neuron's transfer function relates to the relationship between the input and output. The weights and thresholds of the complete neural network are determined by the training function, whereas the weights and thresholds of a single neuron are determined by the learning function. The two are the relationship between global adjustment and local adjustment. A more specific explanation is that the training function uses the overall error to guide the learning function to train local weights and thresholds. The performance function is used to measure the training error and determine whether the training results meet the accuracy requirements.

For example, sigmoid logarithmic function logsig is often employed, as is sigmoid tangent function tansig and linear function purelin. Using the logsig function as a transfer function restricts the network's output value to the range [0, 1]. Using tansig as the transfer function, the network's output value can only be in the interval [−1, 1]. Linear transfer function purelin provides a sufficient output range when it is utilized in the output layer. So the sigmoid function is often used in the hidden layer, while the purelin function is used to broaden the numerical range in the final output. The specialty of BPNN is to deal with linear uncorrelated problems. From a theoretical point of view, the multiarchitecture network layer contains a wealth of independent neurons, and the network model can continuously approximate the function of linear irrelevance. BPNN has the ability to not only collect and process but also upgrade and optimize information. The principle is that its processing method is performed simultaneously in layers, and there is an optimization step in the process of information extraction. During the optimization process, all related neurons operate at the same time. The BPNN can use its own carrying information and optimized processing capabilities to perform self-upgrade operations, but the scattered and fragmented information can also be restored. This characteristic of it can make it fully play in specific fields, such as image acquisition and restoration and language recognition and processing. Finally, the BPNN enables the input information to be screened and summarized by itself. This feature of dealing with uncorrelated problems makes it more applicable and more professional. With this feature, neural networks have developed rapidly and solved problems that traditional methods cannot handle in the field. The excellent computing function of BPNN is also a big advantage. Through continuous optimization and adjustment, the BPNN gradually fits the complex nonlinear problems and finally obtains the optimal solution. Its disadvantage is that the optimal solution obtained may be a local optimal solution rather than a global optimal solution.

### 3.2. Intelligent Optimization Algorithm

In order to solve the shortcomings of the traditional gradient descent method, the genetic algorithm (GA) uses the global search method to greatly reduce the possibility of eventually falling into the local optimal solution by continuously eliminating individuals with poor performance. It can not only use the adaptation of the monomer to learn but also complete the learning through the evolution of the population. At the same time, GA also has unique advantages for solving nonlinear problems. Due to its gradient descent basis, the BP algorithm is prone to local optimums due to the randomness of weights and threshold values at the start of the process. In order to enhance the learning quality of BPNN and limit the risk of it slipping into a local optimum solution, GA screens BPNN's weights and thresholds. There is a genetic process for selection and survival in nature that inspired this algorithm. The best offspring, that is, the optimum solution, may be obtained by performing a sequence of operations on biological chromosomal genes using mathematical concepts and computer simulation, mimicking the natural process of biological evolution. The GA has a good global search ability. Compared with the traditional gradient descent algorithm, it is easy to fall into the defect of the local optimal solution. The GA has certain advantages.

#### 3.2.1. Basic Concepts of GA

Since the GA is based on the theory of biological evolution, many concepts of biological evolution are involved in the GA, which will be briefly explained below. (1) The solution space of the problem: the optimal solution and the suboptimal solution in the GA usually exist in the same set, and this set is the solution space. (2) Individuals and populations: the GA selected multiple original individuals as the solution space, and the collection of individuals forms a population, in which each original solution is a single individual. During the algorithm iteration, the number of individuals in the population does not change, and the individuals in it will continue to “evolve.” (3) Chromosome encoding: in order to carry out individual selection, crossover, and mutation, the GA needs to encode each individual in the population, usually encoding the individual chromosomes into gray strings or binary strings. (4) Fitness function: the fitness function is to measure the gap between the individual and the optimal solution. Selecting the appropriate fitness function is very important for solving the optimal solution and optimization algorithm. (5) Selection: the selection step is completed by selecting excellent individuals from the population through the fitness function. (6) Crossover: exchange positions of homologous chromosomes in individual chromosomes to form two new individuals with new chromosomes. (7) Mutation: a new individual is generated by negating some genes on the chromosome, which is a mutation operation.

#### 3.2.2. Characteristics of GA

Compared with the traditional gradient descent algorithm, the GA has the following characteristics: (1) it has global search ability. Using the biological inheritance and survival of the fittest mechanism in the process of simulating the evolution of species, when we use the GA, we do not need to be limited by the limitations of the search space, so as to explore and collect information in a distributed manner in the entire space. (2) Simple operation: genetic operations measure the gap between a single individual and the optimal solution through fitness value, which reduces the frequency of human-computer interaction in the optimization calculation process. (3) The GA has strong robustness and strong self-learning ability and has a good ability to solve most nonlinear problems of the system.

### 3.3. Using GA to Improve BPNN

The BPNN is an algorithm based on the principle of gradient descent. The selection of initial weights and thresholds is randomly generated and lacks scientific basis, which makes the BPNN more likely to fall into the local optimal solution and the global search ability is relatively poor, and the final prediction accuracy is affected. For this problem, the distributed exploration and collection of information by GA can cover the entire space and has the characteristics of global search. This article, therefore, will apply the GA to improve the original BPNN. Screening and optimization are done using GAs in order to address BPNN's initial weight and threshold being randomly generated. To compensate for the BPNN's unpredictable starting weights and thresholds, a GA-BPNN is developed by combining their properties. The GA-BPNN has both the advantages of the nonlinear mapping of the BPNN and the advantages of the global search of the GA, which further improves the prediction accuracy of the BPNN and finally obtains the global optimal solution.

#### 3.3.1. Setting GA to Improve BPNN Parameters

For this paper, we will select the control parameters of the genetic algorithm, and the specific steps are as follows:

(1) Coding setting: in this paper, real number coding will be used as the coding method. Based on the BPNN structure, the calculation formula of the coding length *L* is as follows:
  (4) $$\begin{array}{c} L=i\times k+k\times j+k+j\text{,} \end{array}$$
  where *i* is the number of nodes in the input layer, *k* is the number of nodes in the hidden layer, and *j* is the number of nodes in the output layer.
(2) Determining the population size: at present, there is no uniform standard for the population size, which is usually set at 10–200, and the number of population sizes in this paper is set at 20.
(3) Selection of fitness function: since this paper aims to evaluate the effectiveness of DBT therapy for PTSD, absolute differences between anticipated and actual data are used to calculate an individual's fitness level. Consequently, the lower a fitness, the more accurate their forecast.
(4) Genetic operation setting selection operation: tournament, roulette, and other mechanisms are used in the GA selection process. The fitness ratio is used to choose the roulette approach in this study. It is simpler to find the best solution area if there is a higher possibility of crossovers occurring. Therefore, the crossover probability in this paper is selected as 0.8. Mutation operation: the mutation probability should not be too large in general, so the mutation probability is selected as 0.1 in this paper.
(5) The terminating evolutionary algebra is usually selected between 50 and 100. In this paper, the terminating evolutionary algebra is 50.

#### 3.3.2. Genetic Algorithm Implementation

After selecting the parameters of the GA, this paper will use the GA to optimize the BPNN, as follows:

(1) Randomly initialize the population. There are four pieces to each person's real number string: the connection weights between the input and hidden layers and thresholds for each of these layers, as well as weights for each of these layers' connections to each other. The individual encoding approach uses real number encoding. By calling the subfunction code, 20 groups of codes consisting of 81 real numbers are randomly generated, which together constitute the initial population. All the weights and thresholds of the BPNN are included in the individual. It is possible to construct a neural network whose structure, weights, and thresholds are known once the network's structure is understood.
(2) Calculate the fitness of the population and find the optimal individual. After the initial population is established, the fitness function is selected and used as the basis for optimization search, and the fitness value of each individual in the population is calculated. The calculation formula is
  (5) $$\begin{array}{c} F=k\left(\overset{n}{\underset{i=1}{\sum }}abs\left(y_{i}-a_{i}\right)\right)\text{,} \end{array}$$
  where *n* is the number of network output nodes, *y*_*i*_ is the expected output of the i-th node of the BPNN, *a*_*i*_ is the predicted output of the i-th node, and *k* is the coefficient. The fitness value of 20 groups of individuals is calculated by calling the subfunction, and the optimal individual is selected, that is, the individual with the smallest fitness.
(3) Select an action. Roulette technique, or proportionate selection approach, is used in this study. Each candidate's chance of getting chosen is directly proportional to their fitness.
(4) Crossover operation: the crossover operation method adopts the real number crossover method, that is, the crossover of the kth chromosome *a*_*k*_ and the first chromosome *a*_*l*_ at the *j* position. The operation method is as follows:
  (6) $$\begin{array}{c} \left\{\begin{array}{c} a_{kj}=a_{kj}\left(1-b\right)+a_{ij}b\\ a_{ij}=a_{ij}\left(1-b\right)+{al}_{kj}b \end{array}\right.\text{,} \end{array}$$
  where *b* is a random number between [0, 1].
(5) Mutation operation: select the jth gene *a* of the i-th individual to mutate. The mutation operation method is as follows:
  (7) $$\begin{array}{c} a_{ij}=\left\{\begin{array}{c} a_{ij}+\left(a_{ij}-a_{\max}\right)f\left(g\right)\;r>0.5\\ a_{ij}+\left(a_{\min}-a_{ij}\right)f\left(g\right)\;r\leq 0.5 \end{array}\right.\text{,} \end{array}$$
  where *a*_max_ is the upper bound of gene *a*_*ij*_, *a*_min_ is the lower bound of gene *a*_*ij*_, *g* is the current generation number, and *r* is a random number between [0, 1].
(6) Repeat the previous three steps repeatedly until the set number of iterations is met. Finally, the individual with the best fitness is obtained by decoding, and the new optimal individual is compared with the optimal individual in step (2). By comparison, the final optimal individual is selected, and the data it contains are the optimal initial weights and thresholds of the BPNN, and the optimal weights and thresholds are obtained through reverse decoding.
(7) The GA-BPNN is created by feeding the original BPNN the optimum input and hidden layer connection weights, hidden layer thresholds, hidden layer and output layer connection weights, and output layer thresholds.

### 3.4. Evaluation Index System of DBT Therapy Effectiveness

In this paper, the following indicators were selected to compare the changes before and after DBT therapy, as shown in Table 1.

## 4. Experiment and Analysis

### 4.1. Experimental Data Source

Taking college students in a certain university as the research objects, excluding those with family history of other neuropsychiatric diseases, alcohol dependence, and drug abuse, the cluster sampling method was used to randomly select college students as the subjects. The survey methods were online and offline anonymous self-administered questionnaires. The questionnaire is divided into two parts: one is the self-made questionnaire and the other is the civilian version of the PTSD Symptom Self-Rating Scale. Self-made questionnaires included general demographic characteristics, such as gender and age, as well as the influencing factors of PTSD, including traumatic events, stressful events and their roles in events, and time of exposure. PTSD symptom self-rating scale civilian version, currently used internationally, has been developed according to the fourth edition of the American Diagnostic and Statistical Manual of Mental Disorders diagnostic criteria, a total of 17 items, used to evaluate the three core symptom groups of PTSD, including reexperiencing emotional numbness and hypervigilance. The scale requires the subjects to make an independent assessment based on their actual situation in the past month and uses a 5-point scoring standard to accumulate the total scores to judge the severity of PTSD. Total score criteria are as follows: no obvious symptoms of posttraumatic stress disorder, total score of 17 to 37 points; a certain degree of symptoms of posttraumatic stress disorder, 38 to 49 points; positive total score of PTSD screening ≥50 points. Judgment criteria for different symptom groups are as follows: among the 17 items, those with a symptom score of ≥3 points are regarded as positive; among the three core symptom groups, those with ≥1 positive reexperiencing symptoms are regarded as positive, and those with ≥3 positive avoidance numbness symptoms are regarded as positive. It was judged to be positive, and those with ≥2 positive symptoms of high alertness were judged to be positive. The higher the total score of the scale obtained by summarizing the scores of each item, the greater the possibility of PTSD. International studies suggest that the reference value range should be lowered to 38 points to increase the effectiveness of its diagnosis. Therefore, in this study, the recommended value greater than or equal to 38 points was used as the standard for positive PTSD screening. A total of 596 people were selected in this survey, and 488 valid questionnaires were finally obtained, including 200 males and 288 females. 475 people were 18–25 years old, 10 people were 25–30 years old, and 3 people were over 30 years old. Among them, 485 had traumatic event experience, accounting for 99.39%, and 3 had no traumatic event experience, accounting for 0.61%.

### 4.2. Model Parameter Selection

**Figure 1:**
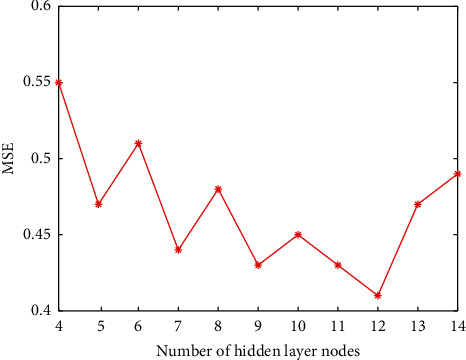
The influence curve of the number of hidden nodes on the training error.

**Figure 2:**
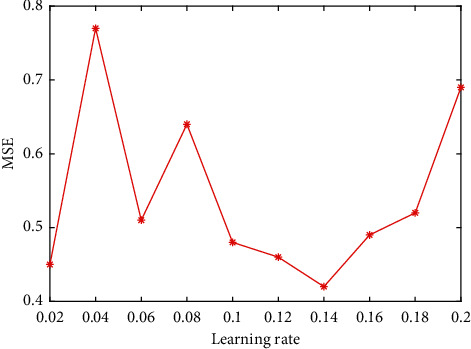
The influence curve of learning rate on training error.

**Figure 3:**
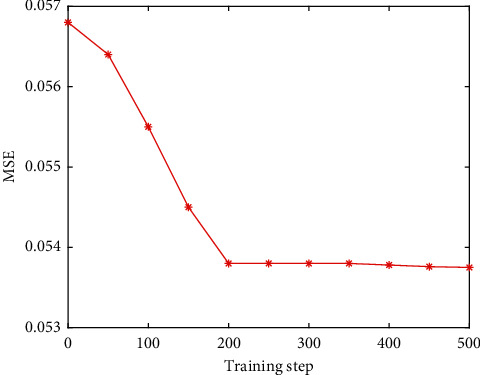
Relationship between training step size and mean squared error.

**Figure 4:**
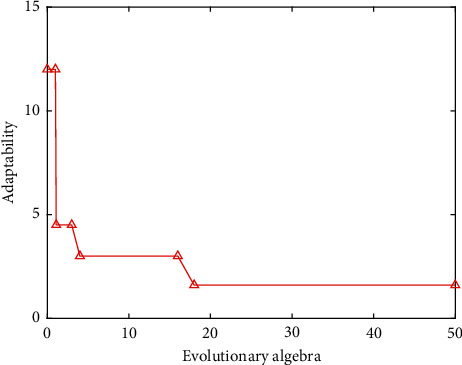
Genetic algorithm fitness curve.

1. Determination of the number of hidden layer nodes: at present, there is no very successful solution for the determination of the number of hidden layer nodes in the theoretical circle. The best method for determining the number of hidden layer nodes is determined according to the experimental results. In this paper, the value range of the number of hidden layer nodes is set as (4, 14), and the network is trained for different node numbers. The experimental results are shown in Figure 1. It can be seen intuitively from Figure 1 that when the number of hidden nodes is 12, the model training error is the smallest, so the number of hidden layer nodes is determined to be 12 in the experiment.
2. Determination of the learning rate: if the learning rate is too large or too small, it will affect the performance of the neural network. In practice, in order to maintain the stability of the network, a slightly smaller learning rate is usually selected. In this paper, the experimental range of the learning rate is set at (0.02, 0.2). After determining the optimal number of hidden nodes, the network is trained with different learning rates in the range. The experimental results are shown in Figure 2.
3. It can be seen intuitively from Figure 2 that when the learning rate increases to 0.14, the training error is reduced to the lowest point, and the errors corresponding to the learning rate in the rest range are too large, so 0.14 is selected as the best learning rate in the network training program.
4. Finally, we compare the training step size. Through programming simulation in Matlab, the relationship between the minimum error and the training step size is studied. The simulation results are shown in Figure 3. The simulation results show that the larger the training step, the smaller the mean square error. When the training step size reaches 200 steps, the training MSE tends to stabilize. Considering the running speed and the efficiency of the simulation experiment, this paper selects the training step size as 200 steps.
5. GA fitness: through the above operations, the sample data is trained, and the fitness function value change curve is obtained as shown in Figure 4.

As shown in Figure 4, the fitness curve dropped sharply between generations 0–18, indicating that the fitness of individuals is increasing. After 18 generations, the fitness curve no longer changes, indicating that the optimal individual has been obtained; that is, the optimal initial weight and threshold have been obtained. The obtained optimal weight threshold is assigned to the GA-BPNN through decoding.

### 4.3. Model Evaluation Accuracy

**Figure 5:**
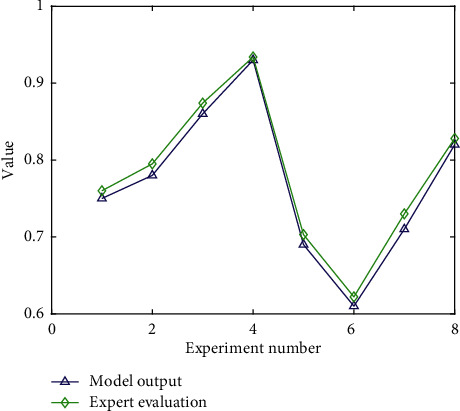
Comparison of model output and expert evaluation results.

In order to verify the accuracy of the model proposed in this paper in evaluating the effectiveness of DBT therapy, this paper compares the output of the model with the evaluation results of psychologists, as shown in Figure 5.

### 4.4. Effectiveness Analysis of DBT Therapy

**Figure 6:**
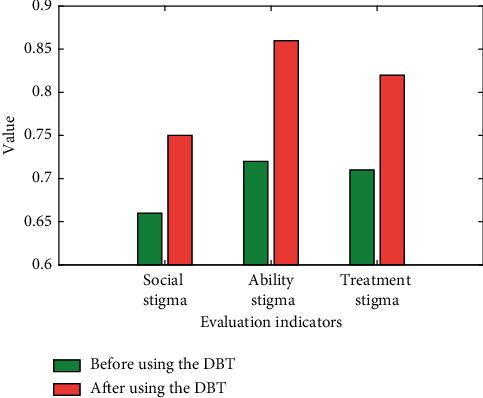
Changes in PTSD indicators before and after DBT therapy.

In order to prove the effectiveness of DBT therapy on PTSD, this paper selected the average of 3 indicators of 10 patients before and after DBT therapy, and the results are shown in Figure 6.

### 4.5. PTSD Risk Factor Analysis

Whether PTSD is positive or not is the dependent variable, general demographic characteristics (gender, age, and whether the parents have died), traumatic events, roles in the traumatic events, and the time of exposure to the traumatic events are independent variables. Multivariate unconditional logistic regression analysis was performed, and the results are shown in Table 2. The results showed that gender, the time of exposure to traumatic events, and whether their parents died may be the main influencing factors of PTSD, and under the same conditions, women are more susceptible to PTSD than men, and the longer the exposure time to traumatic events, the more the susceptibility to PTSD.

## 5. Conclusion

As a mental disorder, PTSD has attracted much attention due to its high incidence, long course, and incurability, which seriously affects people's physical and mental health. Comparatively speaking, students in the university stage are more vulnerable to potential threats. According to statistics, 67% to 85% of those who experienced trauma were exposed to it during this period. As a special group of college students, because their own psychology is not fully mature, and they are facing social transformation and increasingly fierce competition and employment pressure, they are exposed to various stressful or traumatic events, which leads to an increasing trend of the incidence of PTSD. In order to investigate the epidemiological characteristics of PTSD among college students, analyze the possible influencing factors, and achieve the purpose of prevention and intervention, although some explorations have been carried out on the epidemiological research on PTSD in college students, unfortunately, it is limited to the northern part of our country, some colleges and universities in the eastern, southern, and central regions, they did not carry out these studies. Therefore, this study took college students from a certain university as the research object, initially analyzed the epidemiological characteristics of PTSD in college students, and further explored its influencing factors, so as to provide theoretical basis for the prevention and treatment of PTSD and other psychological disorders in college students. And we completed the following work: (1) this paper introduces the research status of PTSD pathogenesis at home and abroad and expounds the treatment methods and research results of DBT. (2) The basic principle of BPNN is introduced, the weight and threshold of BPNN are screened by genetic algorithm, and the best weight and threshold after screening are given to BPNN. A GA-BP model is constructed to improve the learning quality of BPNN. (3) The optimal parameters of the model are selected through experiments, and the model is verified by the collected data. The results show that the model has superiority in evaluating the effectiveness of DBT therapy. Then, it was proved by experiments that DBT therapy has a good effect in the treatment of PTSD. Finally, the influencing factors of PTSD were analyzed one by one through the experimental results.

## Figures and Tables

**Table 1:** DBT therapy effectiveness evaluation index system.

Index	Label
PANSS	T1
Positive	T2
Negative	T3
General psychopathology	T4
SSSI	T5
SQLS	T6
Social stigma	T7
Ability stigma	T8
Treatment stigma	T9

**Table 2:** Logistic regression analysis results of influencing factors of PTSD.

Variable	B	SE	Wald	*P*	OR	95% CI
Gender	−0.831	0.335	6.330	0.021	0.448	0.242∼0.844
Age	−1.243	0.982	1.561	0.222	0.280	0.051∼2.041
Event role	0.117	0.224	0.261	0.625	1.123	0.740∼1.688
Event contact time	0.519	0.168	9.935	0.011	1.657	1.217∼2.258
Parents died	−1.968	0.325	38.437	0	0.150	0.085∼0.271

## Data Availability

The datasets used during the current study can be obtained from the author upon reasonable request.
